# Association between physical activity and sleep disorders in Peruvian schoolchildren: A cross-sectional study

**DOI:** 10.1016/j.sleepx.2025.100160

**Published:** 2025-11-06

**Authors:** Paula L. Arias-Segales, Santos L. Chero-Pisfil, Miguel A. Arce-Huamani

**Affiliations:** a Universidad Norbert Wiener, Lima, Peru; b Programa de Medicina Humana, Facultad de Ciencias de la Salud, Universidad Norbert Wiener, Lima, Peru

**Keywords:** Physical activity, Sleep disorders, Schoolchildren, Pediatric health, Peru

## Abstract

**Background:**

Physical activity and sleep are key determinants of child health, yet evidence from low- and middle-income settings remains limited. We examined the association between physical activity and sleep disorders in Peruvian schoolchildren.

**Methods:**

We conducted a cross-sectional study of 81 children aged 9–12 years from a public school in Callao, Peru (2024). Physical activity was assessed with the Assessment of Physical Activity Levels in Children Questionnaire (APALQ), and sleep disorders with the Tucson Children's Assessment of Sleep Apnea Questionnaire (TuCASA). Given non-normal distributions, associations between total and dimensional activity scores and TuCASA scores were tested using Spearman's rank correlation.

**Results:**

Most participants showed some degree of sleep disturbance; 45.7 % had mild and 17.3 % moderate sleep disorders, while 37.0 % were normal. Regarding activity, 32.1 % were sedentary, 45.7 % moderately active, and 22.2 % very active. Total physical activity was strongly and inversely correlated with sleep disorder scores (ρ = −0.752, p < 0.001). All dimensions type, frequency, duration, and intensity also showed significant inverse correlations with TuCASA scores (all p < 0.001).

**Conclusions:**

Higher physical activity is consistently linked with fewer sleep problems in schoolchildren. Findings support school-based strategies that integrate movement behaviors with sleep health promotion in resource-limited contexts.

## Highlights

• Higher physical activity strongly linked to fewer sleep problems (ρ = −0.752).
• All activity dimensions inversely correlated with sleep disorder scores.
• Validated APALQ and TuCASA instruments used; non-parametric analyses.
• Context-specific evidence from Peruvian schoolchildren in a resource-limited setting.
• Findings support school-based programs integrating movement and sleep health.

## 1 Introduction

Sleep and physical activity are core pillars of child health. Both influence growth, emotion regulation, learning, and metabolic risk [1]. Studies have shown that longer sleep duration and better sleep quality are associated with improved cardiorespiratory and muscular fitness among adolescents, suggesting a beneficial link between healthy sleep patterns and physical fitness [2]. Systematic reviews confirm that school-age children who regularly meet recommended physical activity guidelines report fewer sleep disturbances and better overall well-being [3]. In addition, adequate sleep has been linked to lower prevalence of overweight and obesity, reduced emotional and behavioral problems, and improved cognitive function in childhood [4]. These findings support the importance of promoting both sleep health and physical activity from an early age to ensure optimal growth and long-term health. In summary, current evidence highlights a positive relationship between sleep and physical activity among school-aged populations.

However, several important gaps remain regarding the factors that shape this relationship, especially in low- and middle-income countries with distinct cultural and environmental characteristics [5]. Research indicates that screen exposure, late bedtimes, and insufficient time devoted to structured or spontaneous physical activities are linked to shorter sleep duration and increased sleep disorders among children, yet these factors may differ in importance across diverse settings [6]. The presence of electronic devices in the bedroom, social and family routines, and urban versus rural residence further complicate the understanding of sleep patterns in childhood [7]. Notably, studies focusing on schoolchildren from Latin American countries, such as Peru, are scarce, making it difficult to generalize results obtained in other regions [8]. Furthermore, the few available investigations in South American contexts often rely on parent-reported data or cross-sectional designs, limiting the strength of causal inferences [9]. This lack of region-specific and methodologically robust research creates challenges for the design of effective interventions and public health recommendations tailored to local populations. Understanding these contextual factors is crucial to address disparities and promote healthy behaviors in different communities.

There is also a lack of consensus regarding the directionality and mechanisms of the association between physical activity and sleep disorders among children [10]. While some intervention studies have suggested that increased physical activity improves sleep duration and quality, others report inconsistent results, likely due to variations in measurement tools and study populations [11]. For example, children with neurodevelopmental disorders have been shown to benefit from specific physical activity interventions, but the effect sizes vary depending on activity type, frequency, and duration [12]. Socioeconomic status, family habits, and gender may also moderate the relationship, with some evidence indicating that boys and girls respond differently to physical activity in terms of sleep outcomes [1]. Moreover, few studies have examined how public policies, such as school schedules and community programs, influence the physical activity sleep relationship in diverse educational settings [2]. These unresolved issues underscore the need for population-based studies that can clarify these associations and guide health promotion efforts. A deeper understanding of these complexities will support more targeted and effective strategies for improving child health.

Therefore, the objective of the present study was to establish the association between physical activity and sleep disorders in Peruvian schoolchildren.

## 2 Materials and methods

### 2.1 Study design and setting

This analytical cross-sectional study was conducted at the Pacifico Educational Institution, located in Callao, Peru, during the 2024 academic year. The research was designed using a quantitative approach and a hypothetical-deductive method, focusing on evaluating the association between physical activity and sleep disorders among primary and secondary schoolchildren. The study employed a non-experimental, descriptive-correlational, and cross-sectional design, as data collection occurred at a single point in time without manipulating the study population or variables.

The investigation was approved by the Institutional Ethics Committee of the Universidad Privada Norbert Wiener, and all procedures adhered to the ethical principles outlined in the Declaration of Helsinki and national regulations. Data were collected during the first semester of 2024 through direct administration of standardized questionnaires to the participating students.

### 2.2 Population and sample

The study population consisted of 100 schoolchildren enrolled at the Pacifico Educational Institution. A non-probabilistic convenience sampling strategy was used to select participants who met predefined inclusion criteria. The final analytic sample comprised 81 children aged 9–12 years of both sexes, all of whom regularly attended the institution and provided complete data for the variables of interest. Inclusion criteria were attendance at the Pacifico Educational Institution, aged between 9 and 12 years, and signed informed assent from the child as well as informed consent from their parent or legal guardian. Exclusion criteria included: acute respiratory diseases, use of sleep-inducing medications, diagnosis of attention deficit hyperactivity disorder (ADHD), cardiovascular or metabolic diseases, physical, intellectual, or sensory disabilities, or incomplete questionnaires.

### 2.3 Data collection

Data collection employed the survey technique, using two validated instruments: the Assessment of Physical Activity Levels in Children Questionnaire (APALQ) [13] and the Tucson Children's Assessment of Sleep Apnea Questionnaire (TuCASA) [14]. APALQ (physical activity). We used the Spanish-language Assessment of Physical Activity Levels Questionnaire (APALQ), comprising five items spanning type, frequency, duration, and intensity. Each item has four response options (1–4); item scores were summed to a total score of 5–22. If one item was missing, totals were prorated to the 5-item metric (sum of answered items × 5/number answered), which preserves the 5–22 scale and can yield non-integer totals; therefore, we report medians and IQR with decimals. For descriptive categories we applied thresholds used in Spanish adolescent studies: sedentary (5–10), moderately active (11–16), very active (≥17). We also computed internal consistency for our sample (Cronbach's α = 0.815). These thresholds have been widely used in Spanish-speaking pediatric samples [13,15,16].

TuCASA (sleep problems). We used the Spanish version of the Tucson Children's Assessment of Sleep Apnea (TuCASA) questionnaire (13 items across nocturnal symptoms, snoring, and daytime symptoms; 6-point response scale: Don't know/Never/Rarely/Occasionally/Frequently/Almost always) originally developed in English for children [17,18]. Item responses were summed to a total score (higher values = more symptoms). Formal psychometric validation is available in Brazilian Portuguese; a published Peruvian psychometric validation is not currently available [14]. We applied a Spanish version reviewed by two bilingual clinicians, with a brief forward–back translation check and cognitive debriefing before fieldwork. For descriptive summaries, we present the original TuCASA category bands normal (0–17), mild (18–31), moderate (32–45), severe (46–65) as descriptive strata rather than diagnostic thresholds. Internal consistency in our sample was Cronbach's α = 0.823.

Both instruments were self-administered under the supervision of the research team and with assistance from teachers when necessary, ensuring clarity and comprehension for all participants.

### 2.4 Variables

The main independent variable was physical activity, measured using the Assessment of Physical Activity Levels in Children Questionnaire (APALQ). This validated instrument assesses four dimensions: type of activity, frequency, duration, and intensity. The APALQ consists of five items, each scored on a Likert-type scale, with a total score ranging from 5 to 22. Based on established cut-off points, physical activity was categorized as sedentary (5–10), moderately active (11–16), and very active (≥17). The operationalization of the variable included dimension-specific indicators, such as participation in organized or non-organized sports, frequency and duration of activity, and participation in competitive sports.

The main dependent variable was sleep disorders, evaluated using the Tucson Children's Assessment of Sleep Apnea Questionnaire (TuCASA), with published validation in Brazilian Portuguese. This instrument comprises 13 items grouped into three dimensions: nocturnal symptoms, snoring, and daytime symptoms. Each item is rated on a six-point scale (Don't know, Never, Rarely, Occasionally, Frequently, Almost always), and the total score was categorized for descriptive purposes as normal (0–17), mild (18–31), moderate (32–45), or severe (46–65). The operationalization included assessment of symptoms such as breathing pauses during sleep, daytime sleepiness, and related behavioral or cognitive issues.

Sociodemographic variables included age (in years), sex (male, female), height (cm), and weight (kg), which were used to characterize the sample and to control for potential confounding factors. All variable definitions and categories followed international recommendations.

### 2.5 Data analysis

We first assessed distributional assumptions using the Shapiro–Wilk test. Variables that did not meet normality were summarized as medians (interquartile range, IQR); categorical variables as counts and percentages. Pairwise associations between physical activity and sleep disorders (total TuCASA score and dimensions) were examined using Spearman's rho with exact two-sided p-values. Figures (scatterplots) were produced to visualize associations. All analyses were conducted in Stata 18 (StataCorp, College Station, TX, USA) with α = 0.05. P-values are reported to three decimals; values < 0.001 are presented as p < 0.001 (we avoid “p = 0.000”). We also estimated internal consistency (Cronbach's α) for APALQ and TuCASA in this sample.

Score distributions for APALQ and TuCASA were inspected using histograms with kernel density overlays (Supplementary Figures S1–S2).

### 2.6 Ethical considerations

This study received ethical approval from the Institutional Research Ethics Committee of Universidad Privada Norbert Wiener (CIEI-UPNW), under approval number 0365–2024, dated May 20, 2024. Written informed consent was obtained from all parents or legal guardians, and informed assent was provided by every participating child prior to enrollment. The study was conducted in full compliance with the principles of autonomy, beneficence, non-maleficence, and justice, as outlined in the Declaration of Helsinki and applicable national regulations. Participation was entirely voluntary. Participant confidentiality and anonymity were strictly maintained, and all data were securely stored and used solely for scientific purposes.

Questionnaires were self-administered in classrooms during school hours under the supervision of trained research staff. Seating was arranged to minimize visual access to peers’ responses. Teachers were available nearby to clarify logistical questions but did not stand at desks or oversee individual responses. Completed questionnaires were placed in sealed envelopes, labeled only with study codes, and stored in locked cabinets accessible exclusively to the research team. These procedures were designed to protect privacy and confidentiality during data collection.

### 2.7 Data availability and use of generative AI

All data, questionnaires, and technical documentation used in this study are available from the corresponding author upon reasonable request. No generative artificial intelligence (AI) tools were used in the design, data collection, statistical analysis, or interpretation of this study.

## 3 Results

The study included a total of 81 children aged 9–12 years from the Pacifico Educational Institution. The sample was predominantly female, with 59.3 % (n = 48) girls and 40.7 % (n = 33) boys. In terms of age distribution, most participants were either 9 years old (32.1 %, n = 26) or 12 years old (40.7 %, n = 33), while smaller proportions were 10 years old (21.0 %, n = 17) or 11 years old (6.2 %, n = 5). This distribution reflects a balanced representation across the targeted age groups, with a slight predominance of the oldest and youngest age categories (Table 1).

**Table 1:** Sociodemographic characteristics of the study population.

Characteristic	Frequency	Percentage
Sex		
Male	33	40.7 %
Female	48	59.3 %
Age		
9 years	26	32.1 %
10 years	17	21.0 %
11 years	5	6.2 %
12 years	33	40.7 %

Distribution of participants according to sex and age. The sample consisted predominantly of females and children aged 9 and 12 years.

Regarding physical activity, nearly half of the children (45.7 %, n = 37) were classified as moderately active according to the APALQ, while 32.1 % (n = 26) were considered sedentary and 22.2 % (n = 18) were very active. As for sleep, the TuCASA questionnaire revealed that most participants experienced some degree of sleep disturbance: 45.7 % (n = 37) were classified as having mild sleep disorder, 17.3 % (n = 14) had a moderate sleep disorder, and only 37.0 % (n = 30) were classified as having normal sleep. Thus, most children in this population were moderately active and exhibited mild sleep disturbances (Table 2) (see Table 3).

**Table 2:** Distribution of physical activity and sleep disorder levels.

Category	Frequency	Percentage
Sedentary	26	32.1 %
Moderately active	37	45.7 %
Very active	18	22.2 %
Normal sleep	30	37.0 %
Mild sleep disorder	37	45.7 %
Moderate sleep disorder	14	17.3 %

Distribution of levels of physical activity and sleep disorders. Most children were moderately active and presented mild sleep disturbances.

**Table 3:** Normality tests for main variables.

Variable	Test	Statistic	p-value
Physical activity (APALQ)	Shapiro-Wilk	0.967	0.034
Sleep disorders (TuCASA)	Shapiro-Wilk	0.935	<0.001

Both variables failed the normality test (p < 0.05); therefore, non-parametric statistical methods were used.

The distributions of APALQ and TuCASA total scores are shown in Supplementary Fig. S1–S2. Both exhibited right-skewed shapes, and no severe TuCASA category was observed in this sample.

**Fig. 1:**
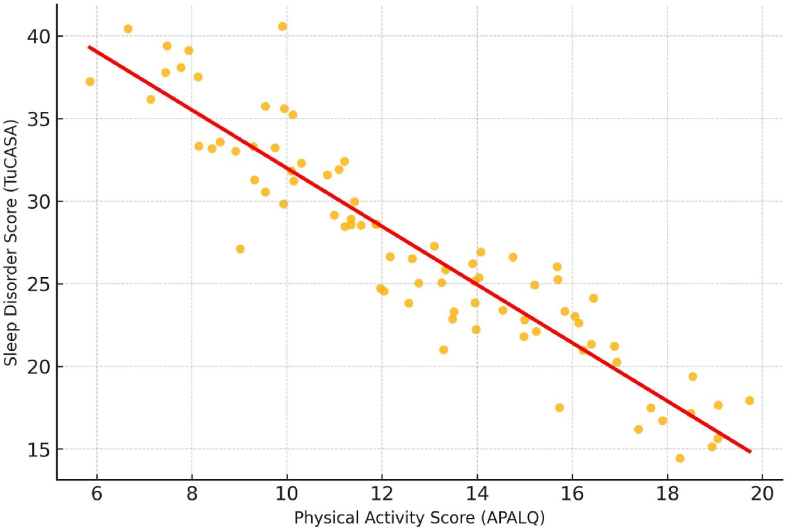
Scatterplot of physical activity (APALQ) vs. TuCASA score A strong inverse correlation was observed (Spearman's rho = −0.752, p < 0.001). A locally weighted smoothing line is included to aid visualization.

A scatterplot was generated to visualize the association between total physical activity and sleep disorder scores. A strong inverse relationship was observed, with higher physical activity levels corresponding to lower sleep disorder scores (Spearman's rho = −0.752, p < 0.001). This negative correlation indicates lower TuCASA sleep problem scores among children with higher physical activity, without implying causality (Fig. 1).

Further analysis examined the specific dimensions of physical activity (type, frequency, duration, and intensity) and their correlations with sleep disorders. All dimensions showed statistically significant negative correlations with sleep disorder scores. Specifically, the Spearman's rho coefficients were −0.477 for type of activity, −0.561 for frequency, −0.470 for duration, and −0.504 for intensity (all p < 0.001). These findings suggest that not only overall physical activity but also component participation in diverse activities, regularity, sustained duration, and higher intensity associated with reduced sleep disturbances among children (Table 4).

**Table 4:** Spearman correlation between physical activity dimensions and sleep disorders.

Dimensions of Physical Activity	Spearman rho	p-value
Total physical activity	−0.752	<0.001
Type of activity	−0.477	<0.001
Frequency of activity	−0.561	<0.001
Duration of activity	−0.470	<0.001
Intensity of activity	−0.504	<0.001

All physical activity dimensions were inversely correlated with TuCASA scores, consistent with fewer reported sleep problems among more active children.

Given the non-normal distribution of both the APALQ and TuCASA scores, descriptive statistics are reported as medians and interquartile ranges (IQR). The median physical activity score was 13.71 (IQR: 11.56–16.01; range: 5–21), while the median sleep disorder score was 24.52 (IQR: 18.68–28.32; range: 10–44). These values further support the observed trends of moderate activity and mild sleep disturbance in the study population (Table 5).

**Table 5:** Descriptive statistics of total scores.

Variable	Median	IQR	Range
Physical activity (APALQ)	**13.71**	**11.56–16.01**	**5–22**
Sleep disorders (TuCASA)	**24.52**	**18.68–28.32**	**10–44**

Since both variables were not normally distributed, medians and interquartile ranges (IQR) were reported along with observed minimum and maximum values.

## 4 Discussion

The present study revealed a high prevalence of sleep disturbances among Peruvian schoolchildren, with nearly two-thirds exhibiting either mild or moderate sleep disorders, and only a minority maintaining normal sleep patterns. At the same time, a substantial proportion of participants were classified as sedentary or only moderately active, with less than a quarter achieving very active status. Most notably, we observed a strong and statistically significant inverse association between physical activity and sleep disorder scores, indicating that higher levels of physical activity were consistently linked to better sleep quality. This relationship persisted across all dimensions of physical activity including type, frequency, duration, and intensity suggesting that both overall engagement and specific activity patterns contribute meaningfully to sleep health in this population. These findings highlight the pressing need to address both insufficient physical activity and sleep disturbances among school-aged children, underscoring their interdependence as critical components of pediatric health and development.

The first key finding of this study is the high prevalence of sleep disorders among Peruvian schoolchildren, with most participants exhibiting some degree of disturbance and only a minority demonstrating normal sleep patterns. When contrasted with the available literature, direct comparisons are limited, as several recent studies do not report explicit prevalence figures for sleep disorders. For example, Larrinaga-Undabarrena et al. [19] and Rodríguez-Negro et al. [20] focused on sleep efficiency and duration, respectively, rather than prevalence. However, Teh et al. [21] reported that 68.2 % of schoolchildren failed to meet age-specific sleep duration recommendations, highlighting a similarly high frequency of insufficient sleep within this population. Likewise, Xiang et al. [22] found that only 64.8 % of children achieved the recommended sleep duration, indicating that sleep insufficiency is widespread internationally, though not all studies use the same operational definitions. Xiao et al. [23] referenced prevalence rates from meta-analyses in China, showing considerable variability across educational levels, ranging from 7.5 % to 41.9 %, further illustrating that methodological differences and local contexts strongly influence reported rates. Overall, while definitions and measurements differ, the consistent finding across regions is that sleep disorders or insufficient sleep are highly prevalent among school-aged children, underscoring the urgent need for targeted interventions and policies that address sleep health as a public health priority in diverse settings.

The second principal result of this study is the relatively low proportion of highly active children, with a substantial segment of the sample classified as sedentary or only moderately active. This pattern is echoed in several recent studies, although direct comparisons are complicated by differences in measurement tools and cut-off points. Larrinaga-Undabarrena et al. [19] reported that 66.94 % of their participants engaged in organized sports, indicating a majority with at least some level of structured physical activity. In contrast, Teh et al. [21] found that 59.1 % of schoolchildren exhibited low levels of physical activity according to PAQ-C criteria, closely mirroring the high prevalence of sedentarism observed in our sample. Xiang et al. [22] provided further support, noting that only 22.6 % of children met the World Health Organization's recommendation of at least 60 min of daily activity, with the remainder falling short of a finding that aligns with the predominance of insufficiently active children in our study. Conversely, Ávila-García et al. [24] reported that 97 % of children in their Spanish sample met activity guidelines, a stark contrast that may reflect cultural, environmental, or methodological differences, including the use of accelerometry versus self-report measures. Rodríguez-Negro et al. [20] presented PAQ-C scores rather than prevalence categories, finding generally low-to-moderate activity levels, with boys being more active than girls. Together, these findings highlight the widespread nature of low physical activity among schoolchildren in many settings and reinforce the necessity of policies promoting active lifestyles to mitigate future health risks.

A central finding of this study was the strong negative association between overall physical activity and the presence of sleep disorders in schoolchildren, indicating that higher levels of activity are closely linked to better sleep health. This relationship finds support in several recent publications, though the magnitude and underlying mechanisms may differ. Teh et al. [21] identified a significant negative association between physical activity (as measured by PAQ-C) and sleep problems (B = −2.139, 95 % CI: −3.357 to −0.921, p < 0.01), which is consistent with our observed robust correlation. Similarly, Xiao et al. [23] found a significant inverse correlation between total physical activity and sleep disorders in adolescents (r = −0.169, p < 0.001), although this association was fully mediated by anxiety and mobile phone dependence, suggesting psychosocial factors may partly explain the relationship. Ávila-García et al. [24] reported that higher daytime activity, both light and moderate-to-vigorous, was associated with improved sleep efficiency, though paradoxically with shorter sleep duration. Larrinaga-Undabarrena et al. [19] also observed greater sleep efficiency in children engaged in organized sports but did not provide a direct correlation coefficient. Alnawwar et al. [25], through a systematic review, concluded that regular physical activity improves sleep quality and reduces sleep disorder prevalence, particularly when exercise is of moderate rather than vigorous intensity. While methodological approaches and outcome definitions vary, the convergence of evidence supports the protective role of physical activity on sleep, emphasizing its public health relevance for childhood well-being.

The analysis of specific dimensions of physical activity such as type, frequency, duration, and intensity revealed that each was negatively correlated with sleep disorders among the children in this study. When examining this pattern considering the recent literature, both similarities and methodological nuances emerge. Teh et al. [21] demonstrated that the PAQ-C score, which captures intensity, frequency, and type of activity, was significantly and negatively associated with several sleep-related subscales, including resistance to sleep (B = −0.068), sleep behavior (B = −0.091), and daytime sleepiness (B = −0.126). This multidimensional association supports our findings that multiple aspects of activity contribute to sleep health. Similarly, Ávila-García et al. [24] found that both light activity and moderate-to-vigorous physical activity were linked to greater sleep efficiency and reduced duration, with stronger effects observed in girls. Xiao et al. [23] reported a significant negative correlation between total physical activity score which integrates frequency, intensity, and duration and sleep disorders (r = −0.169, p < 0.001), although did not analyze the contribution of each dimension separately. In contrast, Larrinaga-Undabarrena et al. [19] provided detailed breakdowns of activity types but did not report explicit correlations with sleep outcomes for each dimension. These results collectively highlight the importance of addressing all components of physical activity in interventions to improve pediatric sleep, as multifaceted activity profiles appear to offer greater protective benefits.

The demographic distribution in this study, characterized by the predominance of girls and most participants aged nine and twelve, presents both parallels and contrasts with recent literature on school-aged populations. Larrinaga-Undabarrena et al. [19] described an almost equal sex distribution (50.1 % boys, 49.9 % girls) and found that primary school children generally slept longer and were less sedentary compared to adolescents, while sleep efficiency was higher among older participants. Rodríguez-Negro et al. [20] also reported a balanced sample (114 boys, 122 girls) and noted that boys were more physically active but had shorter sleep duration than girls, though no significant differences in age or BMI were found between sexes. Teh et al. [21] observed a near-even gender split (48.6 % boys, 51.4 % girls), with boys engaging in more physical activity but showing no significant sex-based differences in sleep parameters. Similarly, Xiang et al. [22] reported a large, balanced sample (51.5 % boys, 48.5 % girls), and documented that adolescents had a higher prevalence of both insufficient sleep and physical inactivity compared to younger children. Although variations in demographic profiles exist between studies, the recurring observation is that sex and age differences subtly influence patterns of physical activity and sleep, underscoring the necessity for age- and gender-sensitive approaches in promoting healthy lifestyles among children and adolescents.

The overall trends observed in this study marked by suboptimal physical activity and high prevalence of sleep disorders are echoed in several recent investigations, though some nuanced differences are notable. Larrinaga-Undabarrena et al. [19] highlighted that children engaged in organized sports not only achieved better sleep efficiency but also exhibited lower sedentary behavior, with adolescents being particularly vulnerable to reduced sleep duration and increased sedentarism. This aligns with our finding of widespread insufficient activity and sleep issues, particularly among certain demographic groups. Rodríguez-Negro et al. [20] similarly reported generally low-to-moderate physical activity (PAQ-C 2.54 ± 0.50) and adequate sleep duration in most participants, though boys were both more active and had shorter sleep than girls, and no significant correlation was found between activity levels and sleep habits. Teh et al. [21] identified that two-thirds of children failed to meet recommended sleep durations and documented a negative association between physical activity and sleep problems, with girls being less physically active but paradoxically at lower risk for sleep disturbances. These findings suggest that while the interplay between physical activity and sleep is complex and influenced by gender and age, the coexistence of low activity and high sleep problems is increasingly prevalent. Addressing these interconnected issues through integrated, context-specific interventions should be a public health priority to improve the well-being of school-aged children globally.

The present study provides valuable evidence on the robust association between physical activity and sleep disorders among Peruvian schoolchildren, underscoring critical clinical and public health implications for pediatric populations. These results highlight the urgent need to routinely assess and address sleep problems in primary care and school health settings, where early detection and prevention are most feasible. By demonstrating that increased physical activity is associated with better sleep health, our findings support the incorporation of physical activity promotion as a cost-effective preventive and therapeutic strategy within pediatric care. Importantly, this study adds to the scarce literature from Latin America and other low-resource settings, contributing novel data from an understudied region where the burden of both inactivity and sleep disturbances is high. In the broader context of a global epidemic of childhood physical inactivity and sleep problems, these findings resonate with international recommendations from the World Health Organization, the American Academy of Sleep Medicine, and leading pediatric societies, all of which advocate for regular activity and healthy sleep routines as cornerstones of child health. Our data reinforce and complement these guidelines, suggesting their relevance extends to diverse and disadvantaged contexts. By giving visibility to Peruvian children an often-overlooked population this research offers valuable insights that may be extrapolated to other developing countries, supporting efforts to reduce health inequities through school-based and community interventions.

This study used validated questionnaires to capture multiple dimensions of physical activity and a comprehensive pediatric sleep symptom profile, enabling a nuanced description of their cross-sectional association. However, several limitations must be acknowledged. First, the sampling strategy was a non-probability convenience sample drawn from a single urban school; the sample is not statistically representative, and external validity beyond similar urban settings is limited. Second, the cross-sectional design precludes causal inference. Third, reliance on self-reported questionnaires may introduce recall and social-desirability biases. Fourth, analyses were bivariate and did not adjust for potential confounders (e.g., age, sex, BMI, socioeconomic status, screen time, mental health), raising the possibility of residual confounding. Finally, some relevant exposures were not measured in detail. These caveats advise cautious interpretation. Future studies in Peru and Latin America should employ probability sampling, longitudinal designs, and multivariable models, and test school-based interventions to clarify causal pathways and policy-relevant effects across diverse urban and rural populations.

## 5 Conclusion

In conclusion, this study established a strong and statistically significant inverse association between physical activity and sleep disorders in Peruvian schoolchildren. Higher levels of physical activity including all its dimensions: type, frequency, duration, and intensity were consistently linked to lower sleep disorder scores and better sleep quality. These findings reinforce the critical interplay between physical activity and sleep health in pediatric populations and highlight the need for integrated, school-based interventions to promote active lifestyles and improve sleep outcomes. By providing robust, context-specific evidence from a Latin American setting, our results contribute to the global understanding of child health and underscore the importance of adopting comprehensive strategies for early prevention and health promotion in school-aged children.

## CRediT authorship contribution statement

**Paula L. Arias-Segales:** Writing – review & editing, Writing – original draft, Investigation, Data curation, Conceptualization. **Santos L. Chero-Pisfil:** Writing – review & editing, Writing – original draft, Investigation, Data curation. **Miguel A. Arce-Huamani:** Writing – review & editing, Validation, Supervision, Methodology, Formal analysis, Conceptualization.

## Ethics approval and consent to participate

The study was approved by the Institutional Committee of Ethics and Scientific Integrity of Universidad Privada Norbert Wiener (CIEIC-UPNW), Approval No. 0365–2024, dated May 20, 2024. Written informed consent was obtained from parents or legal guardians, and child assent was obtained from all participants.

## Declaration of generative AI and AI-assisted technologies in the writing process

No generative AI or AI-assisted tools were used in conception, data collection, analysis, or manuscript writing beyond standard grammar checks.

## Funding

This research did not receive any specific grant from funding agencies in the public, commercial, or non-profit sectors.

## Declaration of competing interest

The authors declare that they have no known competing financial interests or personal relationships that could have appeared to influence the work reported in this paper.

## Data Availability

De-identified data, questionnaires, and technical documentation are available from the corresponding author upon reasonable request.
