# Supplementary material for: Association between physical activity and sleep disorders in Peruvian schoolchildren: A cross-sectional study
Source: Sleep Med X. 2025 Nov 6;10:100160. doi: 10.1016/j.sleepx.2025.100160 (PMC12651353; doi:10.1016/j.sleepx.2025.100160)
Supplement: Multimedia component 1 [file mmc1.docx]

**
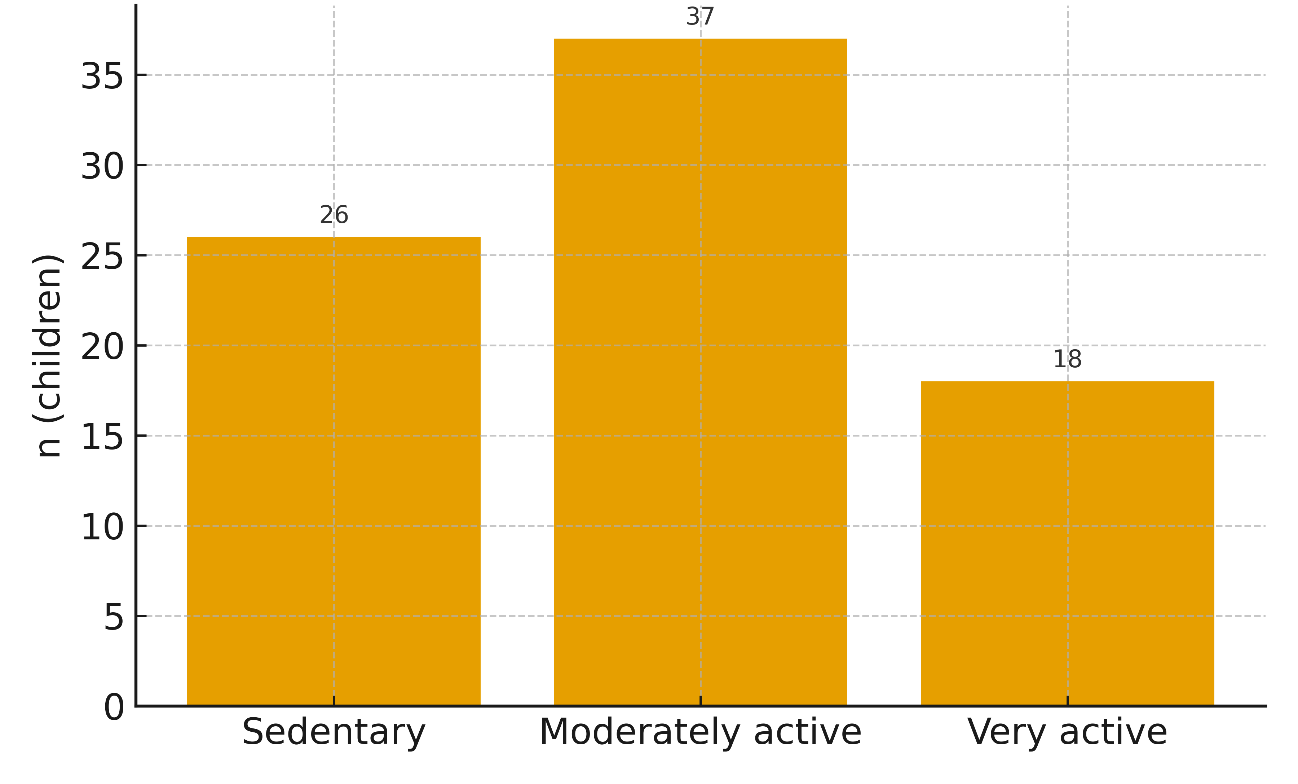
**

**Supplementary Figure S1. Distribution of APALQ total scores.**

Histogram with integer-aligned bins and kernel density overlay. APALQ is the 5-item summed scale (range 5–20).


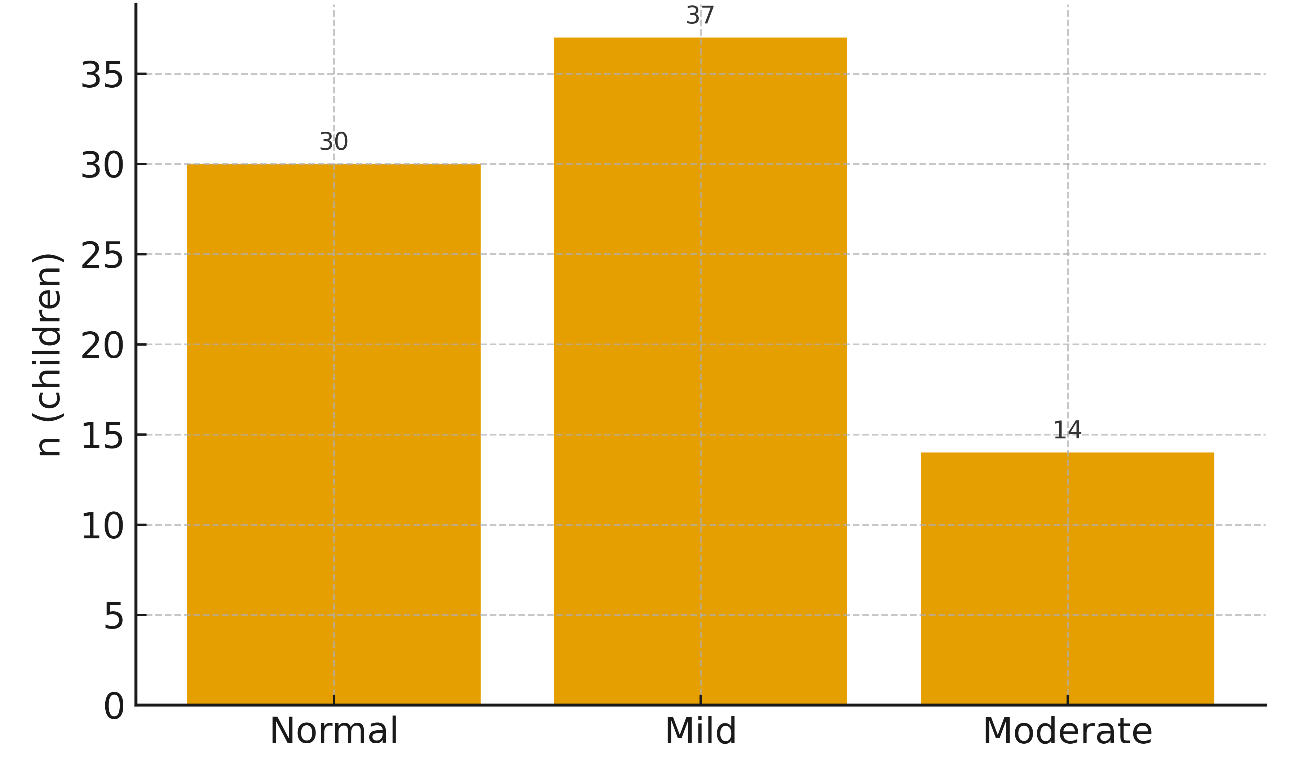


**Supplementary Figure S2. Distribution of TuCASA total scores.**

Histogram with kernel density overlay and vertical reference lines at the TuCASA category cut-points: 18 (normal→mild), 32 (mild→moderate), and 46 (moderate→severe). Avoid color-dependent cues.
